# Influence of Pre-Analytic Conditions on Quantity of Lymphocytes

**DOI:** 10.3390/ijms241713479

**Published:** 2023-08-30

**Authors:** Undine Proschmann, Puya Shalchi Amirkhiz, Pauline Andres, Rocco Haase, Hernan Inojosa, Tjalf Ziemssen, Katja Akgün

**Affiliations:** Center of Clinical Neuroscience, Department of Neurology, University Hospital Carl Gustav Carus, University of Technology, 01307 Dresden, Germany; undine.proschmann@uniklinikum-dresden.de (U.P.); hernan.inojosa@uniklinikum-dresden.de (H.I.); tjalf.ziemssen@uniklinikum-dresden.de (T.Z.)

**Keywords:** autoimmune diseases, flowcytometry, lymphocytes, pre-analytic conditions, storage temperature, storage time

## Abstract

Lymphocytes are key players in the pathogenesis of multiple sclerosis and a distinct target of several immunomodulatory treatment strategies. In this study, we aim to evaluate the effect of various pre-analytic conditions on immune cell counts to conclude the relevance for clinical implications. Twenty healthy donors were assessed for the effects of distinct storage temperatures and times after blood draws, different durations of tourniquet application, body positions and varying aspiration forces during blood draws. Immune cell frequencies were analyzed using multicolor flowcytometry. While storage for 24 h at 37 °C after blood draws was associated with significantly lower cell counts, different durations of tourniquet application, body positions and varying aspirations speeds did not have significant impacts on the immune cell counts. Our data suggest that immune cell counts are differently affected by pre-analytic conditions being more sensitive to storage temperature. Pre-analytic conditions should be carefully considered when interpreting the laboratory values of immune cell subpopulations.

## 1. Introduction

Lymphocytes form the core of the adaptive immune system and represent key players in the pathogenesis of multiple sclerosis (MS). MS is the most common non-traumatic disabling disease affecting young adults [1]. It is known as immune-mediated disease of the central nervous system, characterized by inflammatory demyelination with axonal transection. T- and B-cells as well as cells of the innate immune system are involved in the pathogenesis of MS [2,3,4]. Early MS lesions demonstrate patient-dependent immunopathological heterogeneity in patterns of demyelination [5]. The landscape of disease modifying agents for MS has changed significantly during the last two decades. Many of these new therapeutic approaches target lymphocytes and require frequent evaluation of blood cell counts to monitor treatment effects and to focus on potential treatment-limiting factors [6,7,8]. Beside lymphocyte counts, the characterization of immune cell phenotype is an important element to address basic research questions, to investigate the mechanism of action of emerging immune therapies and to understand individual responses to immunotherapy, particularly in a personalized therapeutic approach [9,10]. Personalized treatment in autoimmune diseases including MS aims to address the individual heterogeneity of the underlying pathophysiological processes, clinical features, prognosis and treatment response. Different factors including order and workflow of blood draw as well as blood storage conditions may interfere with the exact interpretation of individual laboratory results in patients but are of relevance in treatment monitoring and decisions [11,12,13]. However, the impact of sample handling in the pre-analytical phase remains still quite unknown. In this pilot study, we investigated the impact of different blood storage conditions, tourniquet application time, body posture and aspiration speed during blood draws on the immune cell frequencies of healthy donors (HDs).

## 2. Results

Participant’s characteristics are reported in Table 1.

### 2.1. Storage Temperature and Duration

**Figure 1:**
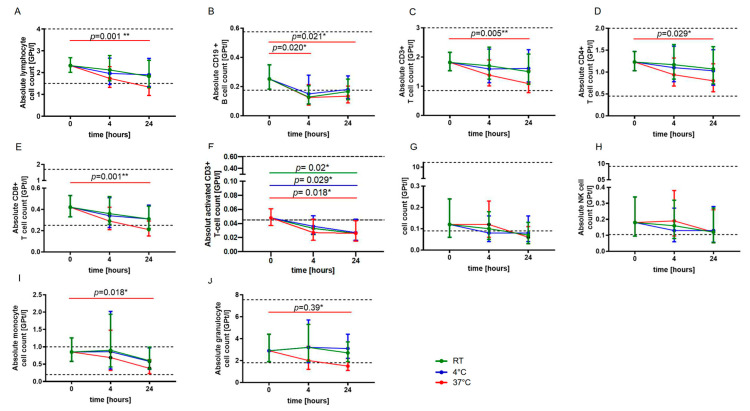
Effect of storage temperature and time on immune cell subsets. The mean absolute cell counts of lymphocytes (**A**), CD19+ B-cells (**B**), T cells (**C**–**F**), NKT cells (**G**), NK cells (**H**), monocytes (**I**) and granulocytes (**J**) with 95% confidence interval (CI) at baseline (0) and after four and 24 h of storage at RT (22 °C) (green, *n* = 5), 4 °C (blue, *n* = 5) and 37 °C (red, *n* = 5) are presented. For each time point, an independent experiment was performed. The reference range is defined by dashed lines. The data were analyzed using generalized linear mixed models for repeated measures. The asterisks indicate a statistically significant difference (* *p* < 0.05, ** *p* < 0.01). For each time point, an independent experiment was performed.

For blood sample storage at RT and 4 °C, no statistically significant changes in absolute cell counts of lymphocytes, CD3+ T cells, CD4+ and CD8+ T cells, monocytes as well as granulocytes were observed after 24 h, whereas a significant decrease in absolute cell numbers was revealed for storage at 37 °C (Figure 1A,C–E,I,J). The absolute CD19+ B cell count was significantly reduced already after four hours of storage at 37 °C (Figure 1B). The absolute activated T cell (CD3+ HLADR+) count was found to be significantly reduced after 24 h independent of storage temperature (Figure 1F). No significant changes were detected for natural killer (NK) T cells and NK cells (Figure 1G,H).

### 2.2. Tourniquet Application, Body Posture and Aspiration Speed

**Figure 2:**
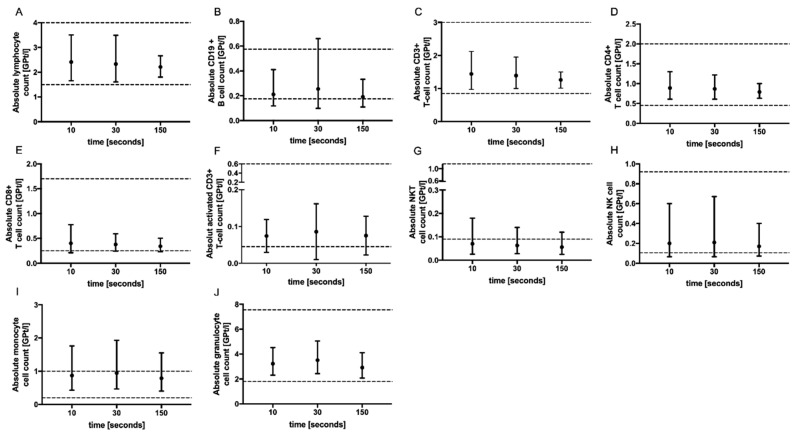
Effect of tourniquet application time on immune cell subsets. The mean absolute cell counts of lymphocytes (**A**), CD19+ B-cells (**B**), T cells (**C**–**F**), NKT cells (**G**), NK cells (**H**), monocytes (**I**) and granulocytes (**J**) with 95% CI after tourniquet application for 10 (*n* = 5), 30 (*n* = 5) and 150 (*n* = 5) s are presented. For each tourniquet application time, an independent experiment was performed. The reference range is delimited by dashed lines. The data were analyzed using generalized linear mixed models for repeated measures. For each time point, an independent experiment was performed.

**Figure 3:**
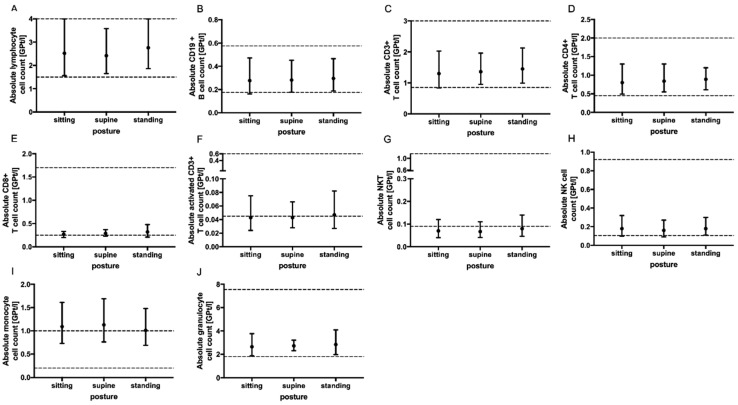
Effect of body posture on immune cell subsets. The mean absolute cell counts of lymphocytes (**A**), CD19+ B-cells (**B**), T cells (**C**–**F**), NKT cells (**G**), NK cells (**H**), monocytes (**I**) and granulocytes (**J**) with 95% CI related to patient posture (sitting *n* = 5, supine *n* = 5 and standing *n* = 5) during blood collection via venipuncture are presented. The blood samples were collected ten minutes after postural change. For each body posture, an independent experiment was performed. The reference range is delimited by dashed lines. The data were analyzed using generalized linear mixed models for repeated measures. For each time point, an independent experiment was performed.

**Figure 4:**
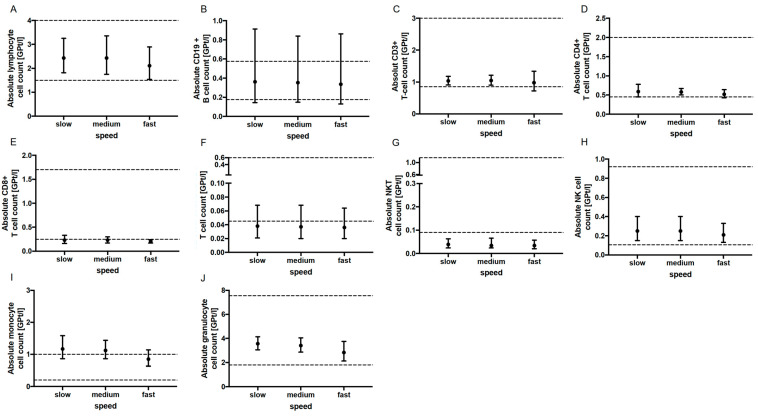
Effect of aspiration speed on immune cell subsets. The mean absolute cell counts of lymphocytes (**A**), CD19+ B-cells (**B**), T cells (**C**–**F**), NKT cells (**G**), NK cells (**H**), monocytes (**I**) and granulocytes (**J**) with 95% CI after slow (*n* = 5), medium (*n* = 5) and fast (*n* = 5) aspiration during blood collection are presented. Lymphoytes For each aspiration speed, an independent experiment was performed. The reference range is delimited by dashed lines. The data were analyzed using generalized linear mixed models for repeated measures. For each time point, an independent experiment was performed.

No significant change in the absolute cell counts of lymphocytes, CD3+ T cells, CD4+ and CD8+ T cells, monocytes, granulocytes, CD19+ B cells, activated T cells (CD3+ HLADR+), NK T cells as well as NK cells could be demonstrated with prolonged tourniquet application (Figure 2), postural change (Figure 3) and distinct aspiration speeds (Figure 4).

## 3. Discussion

Immunotargeting therapies as state-of-the-art treatments for MS require frequent monitoring of the peripheral immune status [8,14]. Clinicians rely on accurate laboratory test results for guiding therapy [15]. Lymphopenia is a common side effect of the majority of MS therapeutics. Immune statuses represent the therapeutic response, the compliance of the patient and the occurrence of treatment limiting side effects. Additionally, the monitoring of lymphocyte counts is crucial for therapy sequencing and planning washout periods [16]. So far, it is estimated that more than 70% of clinical decisions are based on information derived from laboratory results. Hence, knowledge about the effect of pre-analytic conditions on blood parameters is relevant for the correct interpretation of findings by clinicians. Although errors can arise during the entire process of blood testing, studies showed that the pre-analytical phase accounts for 46% to 68.2% of errors observed during the total testing process [17]. Immune cell counts and function are influenced by pre-analytic conditions to various degrees. In this study, we present a different response pattern of leukocytes and lymphocyte subsets to different time-to-processing and temperature-before-processing conditions. Whereas the storage of fresh blood samples at 37 °C for 24 h was associated with significant reduced absolute cell counts of lymphocytes, CD3+ T cells, CD4+ T cells, CD8+ T cells, CD19+ B cells, monocytes and granulocytes, no significant change for these cell types was observed for storage at RT and 4 °C. Activated CD3+ T cells seemed to be more sensitive to storage temperature and delayed processing and were found to be markedly reduced after 24 h at all three investigated storage temperatures. NKT and NK cells represent cell populations that were not significantly affected by storage conditions. Our results are in line with those of a recent study by Diks et al. reporting that the storage of whole blood samples in EDTA tubes at 4 °C and RT for up to 72 and 24 h, respectively, is suitable for reliable immunophenotyping [13]. For the incubation of PBMC at 37 °C, another study detected a higher apoptotic rate compared with storage on 4 °C that is comparable with our findings [18]. Recently, a study reported an increase in NK cells and no change in B cells after 4 °C storage before PBMC isolation and demonstrated that NK cytotoxicity was well preserved at 4 °C in acid citrate dextrose but not in heparinized tubes [19]. The loss of cell populations in blood samples stored at 37 °C might be related to several factors. One contributing factor could be a temperature-dependent alteration in the interaction between samples and the storage container surface. In line with other reports, we hypothesis that the cell viability of lymphocyte subsets might be differentially affected by biochemical changes during storage, anticoagulant type and blood specimen container, supposedly due to differences in cellular cytotoxicity and costimulatory signals [20,21].

In general, a delay from blood collection to blood processing should be minimized whenever possible, and the storage conditions should always be adjusted to the main analytical technique and target as the optimal storage conditions may differ for distinct cell populations and characteristics within the same blood sample.

Additional investigated pre-analytical sampling conditions including body posture change and varying aspiration speeds detected no significant effect on the immune cell counts. Our findings for postural change are different from the results that presented a significant increase in leukocytes, neutrophil, lymphocyte and basophile counts after a postural shift from the supine to sitting positions [12]. Lima-Oliveira et al. suggested a minimum period of 15–20 min of resting in the reference position before the collection of blood samples [12]. However, routine blood samples are usually drawn after only a few minutes in the reference position. A prolonged tourniquet application longer than 60 s is well known to be associated with an alteration in the soluble analytes in blood [22,23]. We could not define an increase in leucocyte and lymphocyte subsets for a prolonged tourniquet application up to 150 s.

## 4. Materials and Methods

### 4.1. Subjects

A total of 20 HDs were recruited for the assessment of the impact of pre-analytic conditions including storage time and temperature, tourniquet application, body posture and aspiration speed on immune cell counts. For each pre-analytic condition, five HDs were examined.

### 4.2. Sample Collection

Venous blood was drawn from HDs with a 21-gauge butterfly needle into an ethylenediaminetetraacetic (EDTA) K blood tube (Sarstedt, Nümbrecht, Germany). We analyzed the effects of blood storage at room temperature (RT, range 20–22 °C), 4 °C and 37 °C for 4 and 24 h compared with immediately processed blood samples; of tourniquet application for 10, 30 or 150 s; of body posture (sitting versus supine versus upright standing); and of slow (max 60 s), medium (16–20 s) and fast (10–15 s) blood aspiration. For evaluation of the effects of body position, blood draws were performed 10 min after postural change from sitting to supine or sitting to upright standing. Each blood draw was performed by the same person.

### 4.3. Ethic Approval

This study was performed according to the Declaration of Helsinki and approved by the Ethics committee of the Faculty of Medicine of the Dresden University of Technology, Germany. All participants provided written informed consent.

### 4.4. Immune Cell Phenotyping Using Fluorescence Activated Cell Sorting (FACS)

The samples used to analyze the effects of storage time or temperature were either freshly processed (0 h) or stored for 4 or 24 h at either RT, 4 °C or 37 °C before processing. All other whole fresh blood samples were prepared immediately for immunophenotyping. Cell count per µL was calculated by counting cells in an improved Neubauer counting chamber by an experienced medical technical assistant. For cell counting, the blood samples were diluted with 3% acetic acid solution at a 1:20 ratio. Ten microliters of the prepared dilution was added to the counting chamber. After having placed the Neubauer Chamber on the microscope stage and having focused the microscope, cell counting was performed three times per sample via a zig-zag technique and the average cell count was calculated. A coefficient of variation ≤0.15 between cell counts was accepted. According to the conventional counting principle, cells on the top and the left boundaries were counted, whereas cells on the bottom and the right boundaries were not counted. The four squares placed at the corners were used for white cell counting. The exact volume of one big square was 0.1 mm^3^. Since the leukocytes were counted in four squares, the total volume was 0.4 mm^3^ = 0.4 μL. Considering dilution at a 1:20 ratio (blood in acetic acid), the following formula was applied:

Leukocytes/µL blood = (counted cell number/0.4 μL) × 20

One hundred microliters of whole blood was added to each tube for FACS analysis. The 14 color protocol allowed us to measure all leukocyte subpopulations in two tubes. The immune cell populations were characterized via surface staining with the following fluorescence labeled antibodies according to the manufacturers’ instructions: Tube 1: CD45-FITC (HI30, BD Bioscience, San Jose, CA, USA), CD3 APC Cy7 (SK7, BD Bioscience), CD19-PE Cy5 (HIB 19, BD Bioscience), CD8-PerCP CY5.5 (RPA-T8, BD Bioscience) and CD4-PE Cy7 (RPA-T4, BD Bioscience); Tube 2: CD45-FITC (HI30, BD Bioscience), CD3 APC Cy7 (SK7, BD Bioscience), HLADR-BV510 (G46-6; BD Bioscience, San Jose, CA, USA), CD14-PECF 594 (MφP9, BD Bioscience) and CD56-BV785 (5.1H11, Biolegend). For lysis of the red blood cells, a FACS lysing solution (BD Bioscience) was added. Viability dye (VD, eBioscience, Thermo Fischer Scientific, Waltham, MA, USA) staining was used to evaluate the apoptotic cells. A gating strategy for cells is presented in Supplementary Figure S1. The cell frequencies were evaluated on an LSR Fortessa cytometer (BD Bioscience, San Jose, CA, USA). For calculation of the absolute leukocyte count, all events of the sample were acquired. The percentage of CD45+ cells was set in relation to all events to calculate the absolute leukocyte count. The absolute lymphocyte, monocyte, granulocyte and lymphocyte subset cell counts were calculated by setting the percentage of these cells in relation to the parent cell population.

### 4.5. Statistical Analysis

Normal distribution of the data was visually assessed using quantile–quantile plots and confirmed via a Shapiro–Wilk test. The quantitative population characteristics were presented as measures of central tendency (mean), followed by standard deviation (SD). The data were analyzed by applying the generalized linear mixed model (GLMM) with gamma distribution and a log link function because of the right-skewed distribution pattern of the data and mode (storage time, storage temperature, tourniquet application time, posture and aspiration speed) as a fixed effect and an interaction of storage time and temperature, respectively. *p*-values < 0.05 were considered statistically significant. For pairwise comparisons, contrast tests with Fisher’s least significant difference procedure were applied. Statistical analyses were performed using the IBM SPSS software for MAC (version 25.0, IBM Corportation, Armonk, NY, USA) and GraphPad Prism (version 7; GraphPad Software, La Jolla, CA, USA).

## 5. Conclusions

Although our findings are limited by the small sample size, we could demonstrate that lymphocyte subpopulations are sensitive to storage time as well as temperature. Overall, the T and B cells were mostly affected from prolonged storage at 37 °C whereas the NKT and NK cells were affected by neither storage time nor storage temperature. Since the results of the immunophenotyping are of clinical significance, it is of decisive importance to determine any pre-analytical errors and to avoid them if possible. Both in a monocentric and in a multicenter clinical research setting, standard pre-analytic operating procedures are necessary to obtain reliable results. Our observations should be confirmed and further elucidated in larger studies, aiming for a standardization and validation of pre-analytical parameters.

## Figures and Tables

**Table 1:** Characteristics of healthy donors (*n* = 20).

Pre-Analytic Factor	Age, Years, Mean (SD)	Gender, Female/Male	BMI, Mean (SD)
Storage temperature and time (*n* = 5)	25.4 (2.3)	3/2	24.5 (3.4)
Tourniquet application time (*n* = 5)	23.2 (0.8)	3/2	24.1 (2.9)
Body posture (*n* = 5)	26.4 (5.4)	1/4	26.6 (2.5)
Aspiration speed (*n* = 5)	24.6 (2.4)	3/2	22.8 (2.5) ^1^

Abbreviations: SD, standard deviation; BMI, Body Mass Index; ^1^ body weight and length available for 3/5 healthy donors.

## Data Availability

The datasets generated and analyzed during the current study are available from the corresponding author upon reasonable request.

## Supplementary Materials

The following supporting information can be downloaded at: https://www.mdpi.com/article/10.3390/ijms241713479/s1.
