# Supplementary material for: Influence of Pre-Analytic Conditions on Quantity of Lymphocytes
Source: Int J Mol Sci. 2023 Aug 30;24(17):13479. doi: 10.3390/ijms241713479 (PMC10487632; doi:10.3390/ijms241713479)
Supplement: Supplementary file 1 [file ijms-24-13479-s001.zip › ijms-2553796-supplementary.pdf]

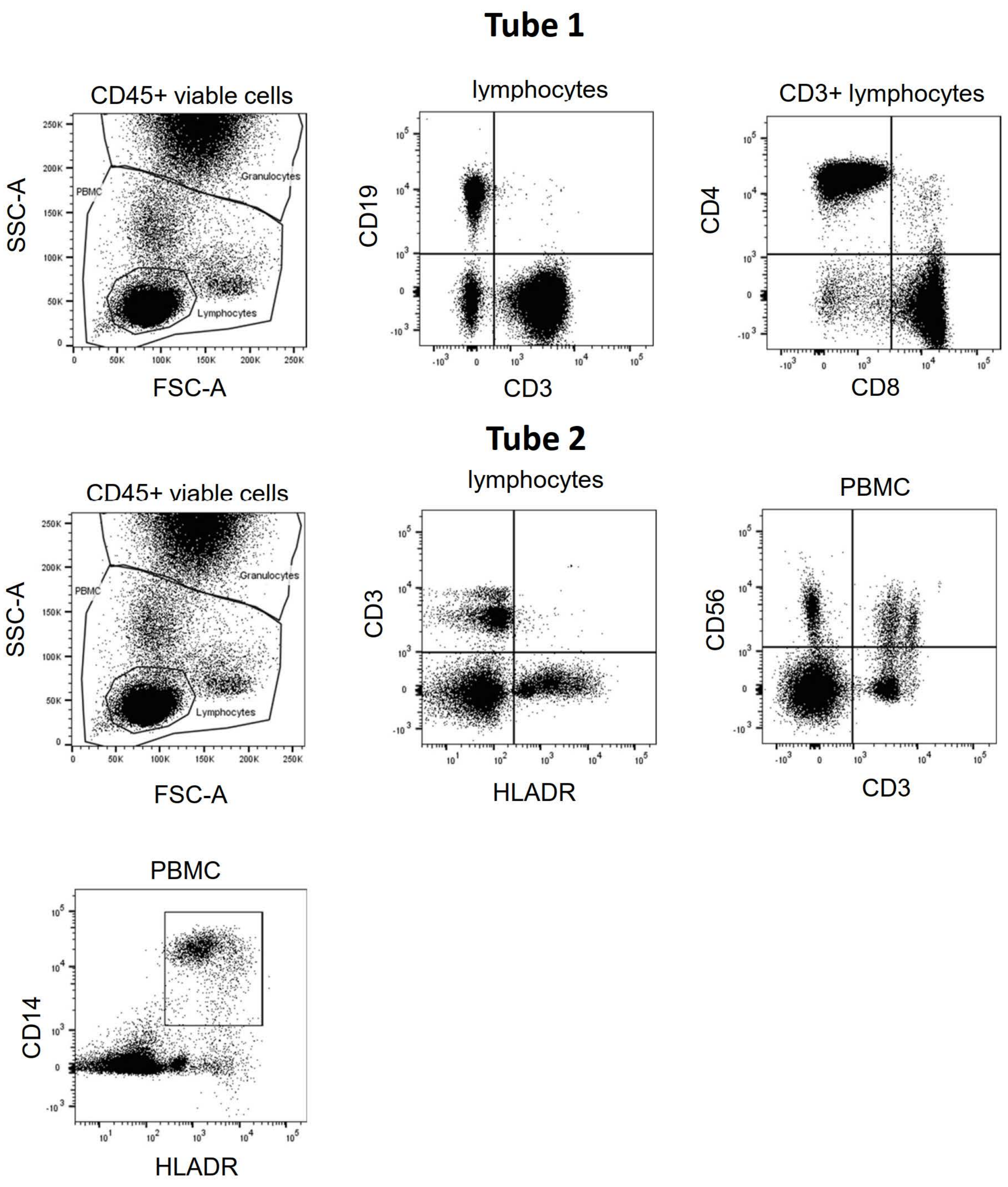

Figure S1. Gating strategy of analyzed peripheral immune cell subsets evaluated by FACS analysis

A hierarchical gating strategy identified single cells using FSC-A versus FSC-H dot plot. Dead cells and non-leukocytes were excluded with fixable Viability Dye (VD) and gating on CD45. Lymphocytes, peripheral blood mononuclear cells and granulocytes were gated based on SSC-A versus FSC-A. Subsequent gating analysis allowed identification of the following subtypes: CD19+ B cells, CD3+ T cells, CD4+ and CD8+ T cells, CD3+ CD56+ NKT cells, CD3+ HLADR+ activated T cells, CD3-CD56+ NK cells and CD14+HLADR+ monocytes.
